# Perceived attitudes of the importance and barriers to research amongst Rwandan interns and pediatric residents – a cross-sectional study

**DOI:** 10.1186/s12909-018-1425-6

**Published:** 2019-01-03

**Authors:** Hubert Habineza, Christian Nsanzabaganwa, Naphtal Nyirimanzi, Christian Umuhoza, Katie Cartledge, Craig Conard, Peter Cartledge

**Affiliations:** 1 University of Rwanda, Kigali, Rwanda; 2 University Teaching Hospital of Butare, Huye, Rwanda; 3 University Teaching Hospital of Kigali, Kigali, Rwanda; 4 Yale University (USA), Rwanda Human Resources for Health (HRH) Program, Kigali, Rwanda

**Keywords:** Medical students, Internship and Residency, Research, Barriers, Attitude, Developing countries

## Abstract

**Background:**

Globally, interns and residents face significant challenges with respect to research activity. Despite this, they are motivated and have an interest in undertaking research. To date, there has been no research regarding the perceived attitudes towards research activities amongst Rwandan residents and interns.

**Objectives:**

The primary objective of this study was to describe the perceived attitudes regarding the educational benefits and barriers surrounding research activity amongst interns and residents, and to identify any differences between these groups. The secondary objective was to describe the research methods used by interns and residents in Rwanda.

**Methods:**

A cross-sectional descriptive study of interns and pediatric trainees at the University of Rwanda. An online questionnaire using Likert scale questions was sent electronically to eligible participants.

**Results:**

A total of sixty participants (38 interns and 22 pediatric residents) responded to the survey. Both groups acknowledged the educational importance of undertaking research, with interns reporting this more than residents. Both groups identified the following as barriers to research: faculty lacking time to mentor, lack of funding, lack of statistical support, and lack of faculty experienced in conducting research. Interns (87%) were much more likely to have undertaken retrospective research than pediatric residents (14%). Few interns or residents submitted their research for publication (27%).

**Conclusions:**

Both interns and residents understood the importance of research, but many barriers exist. Increasing the time available for experienced faculty members to supervise research is challenging due to low faculty numbers. Novel solutions will need to be found as well as expanding the time for trainees to perform research.

## Background

Africa as a continent is still behind the rest of the world regarding the number of centers of excellence in research and the amount of national financial investment going into research and the output of scientific publications [1]. Additionally, frequent regional conflicts adversely influence African research activities due to delays in health sector development and the wastage of countries’ resources [2]. African research should be advanced and strengthened in order to provide the solution to African specific health needs that change over time [1, 3, 4]. In order to do this a better understanding of the perceived attitudes of future researchers is required.

Globally, interns and residents face significant challenges with respect to research activity. In Rwanda, medical students (interns) and residents face various barriers to performing research, notably a lack of reliable high-quality mentorship along with financial burdens. Despite this, they are expected to undertake research in order to graduate from their training at both the undergraduate (interns) and postgraduate (resident) level [5]. It is an aim of the University of Rwanda (UR) to improve the quality and quantity of their research output [6]. In 2014, thanks to intervention from the broader community, Rwanda became the most influential country for published research output in East Africa, with the University of Rwanda (UR) being the second most influential University in East Africa [6].

Globally, medical students (interns) are motivated and have an interest in undertaking research [7–10]. Cited motivations for undertaking research include an interest in the topic, scientific problems, personal or intellectual development, acquisition of critical thinking skills, extra income generation, interest in healthcare development, determination of a specialty/career path, increased interaction with faculty members, and presentation of the work [10, 11]. Involvement of medical students and residents in research is effective in improving their knowledge and research skills [7, 12, 13]. Medical students and residents acknowledge the inherent educational benefits of publishing in peer-reviewed journals. These include; building a Curriculum Vitae, expanding knowledge base within a speciality, and sharing medical discoveries [14].

Many postgraduate (PG) residency programs require residents to undertake a piece of research work as a condition of graduation. The literature suggests that engaging residents in research activities can lead to increased participation in research after residency and increase the number who choose to undertake sub-specialty training [15–17]. The most common identifiable influences on conducting research as a resident are the availability of time, personal interest in research, advanced degrees, future career plans, and availability of opportunities and mentors [18].

There are significant challenges with respect to research activity at an intern and resident level [5, 8, 10, 12], namely; lack of funds and mentorships in the area of research, heavy clinical workload, difficulty in combining medical studies with research, alternative priorities, challenges gaining ethical approval, and a lack of research as a component of the medical curriculum. Funding and mentoring have been found to both increase the number of medical students who may choose to be physician-scientists and improve the quality and quantity of publications undertaken by undergraduate medical students [8, 19, 20].

### Objectives

The primary objective of this study was to describe the perceived attitudes to the educational benefits and barriers regarding research activity amongst interns and residents, and to identify any differences between these groups. The secondary objective was to describe the research methods used by interns and residents in Rwanda.

## Methods

### Study design

A cross-sectional, descriptive survey. Reporting of this study has been verified in accordance with the STROBE (Strengthening the Reporting of Observational Studies in Epidemiology) checklist [21].

### Study setting and location of the population

The University of Rwanda (UR) is a single, multi-campus institution formed from the merging of the nation’s seven public Higher Learning Institutions (HLIs) into a consolidated entity in 2013 [6]. UR comprises of six colleges with the School of Medicine belonging to the College of Medicine and Health Sciences (CMHS). UR is the only institution in Rwanda to offer residency programs and the only institution to have graduated interns. At the time of writing, a new medical school (Gitwe University) has students up to year five, with medical graduates expected in 2019. At the time of the study, UR medical students undertook six years of studies while the majority of MMed (Masters of Medicine) residency programs take four years. All interns and MMed pediatric residents at the University of Rwanda (UR) are required to undertake a research project and write this up as a research dissertation as a condition of graduation [22]. Approximately 75% of medical students in Rwanda are male and this is also reflected in the residency programs.

### Participants/subjects

#### Inclusion criteria

Group 1: Interns who had completed a research dissertation in their final year of medical school (2015–2017), but not yet entered a residency program (*n* = 190). Group 2: Pediatric residents who had completed a residency program between 2012 and 2017 (*n* = 41).

#### Enrolment

Enrolment took place between August and October 2017. Group 1: For interns, we contacted class representatives of the two promotions who sent out the questionnaire link to their classmates via the class WhatsApp group. Group 2: A list of all graduates from the Pediatric Residency program since 2008 is available within the pediatric academic team at the UR. This was used to send a personal email including a link to the online questionnaire.

### Questionnaire and data-collection

The questionnaire was written specifically for this study. The items were drawn from existing studies in the field [8, 11, 23]. The questionnaire was split into four sections: 1. Demographic details and research experience; 2. Educational benefits of research; 3. Developing skills for publishing; 4. Barriers to undertaking research. Sections 2–4 used five-point Likert scales to gain responses. Four senior pediatric academic faculty members at UR reviewed the questionnaires to ensure content validity. The questionnaires were administered online using Google Forms; a free, web-based tool. The Likert scores from the three sections were combined to form “survey scales” (i.e. total composite scores) for comparison. The primary objective was a description of these items and a comparison of the three total section scores.

### Sample size

The three survey scales were non-normally distributed and a post-hoc power calculation was performed, using G*power software, revealing that a 20% difference between the intern and resident groups (alpha = 0.05, power = 0.95) could be identified with the current sample size of 38 interns and 19 residents.

### Statistical analysis

Data was analyzed using SPSS (statistical software for analysis) Version 24. Individual Likert scales were presented as means [24]. The Likert scores from the three sections were combined to form “survey scales” (i.e. total composite scores) for comparison. Responses by residents and interns to individual survey items and survey scales were compared using the Mann-Whitney non-parametric test [25].

## Results

### Data

There were no missing data-points in the data as all questions were “compulsory” in the Google Form. Question responses in Tables 2, 3 and 4 have been presented in order of mean scores rather than order in which presented to participants.

### Baseline details and research experience of participants

**Fig. 1:**
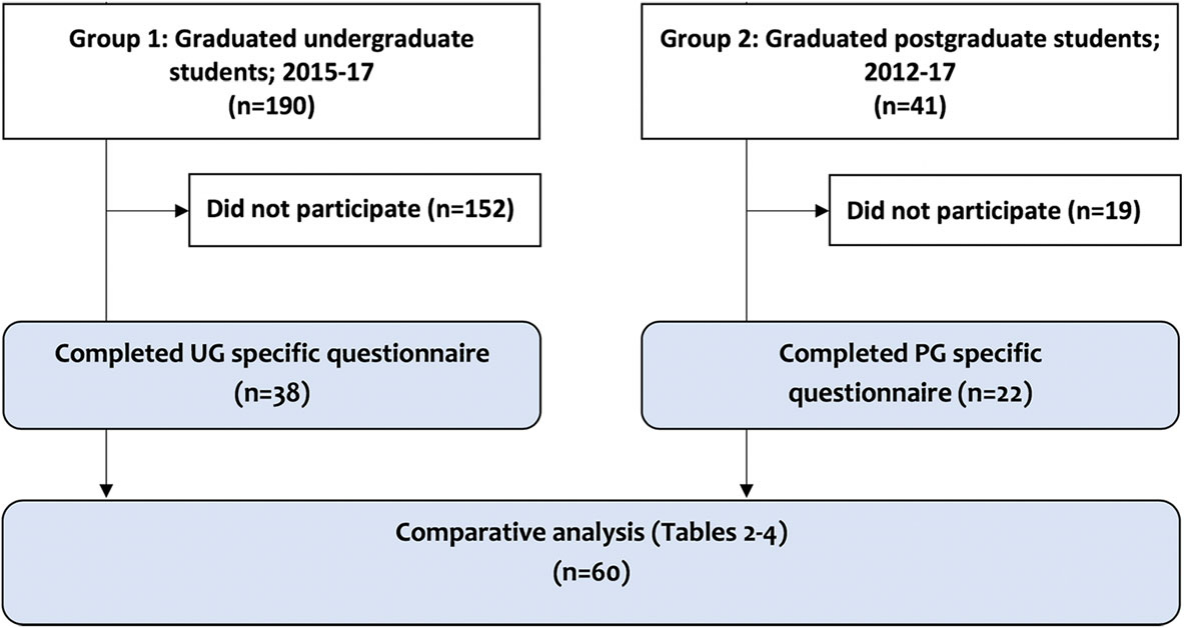
Participant enrolment

We invited 190 interns and 41 graduated pediatric residents to participate with an uptake of 38/190 (20%) and 22/41 (54%) respectively (Fig. 1 & Table 1). The majority of participants were male (75%) with a mean age of 27 and 35 years for interns and residents respectively.

**Table 1:** Baseline details and research experience of participants

	Group 1: Interns (*n* = 38)	Group 2: Residents (*n* = 22)	Total (*n* = 60)
Response rate	38/190 (20.0%)	22/41 (53.7%)	60/231 (30.0%)
Male	27 (71.1%)	18 (81.9%)	45 (75.0%)
Age (mean)	27 years	35 years	30 years
Data collection site			
Tertiary	31 (81.6%)	7 (31.9%)	38 (63.3%)
District	2 (5.3%)	5 (22.7%)	7 (11.7%)
Multi-site	4 (10.5%)	4 (18.1%)	8 (13.3%)
Other	1 (2.6%)	6 (27.3%)	7 (11.7%)
Timing of project			
Retrospective	33 (86.9%)	3 (13.7%)	36 (60.0%)
Prospective	3 (7.9%)	15 (68.1%)	18 (30.0%)
Mixed	1 (2.6%)	2 (9.1%)	3 (5.0%)
Unknown	1 (2.6%)	2 (9.1%)	3 (5.0%)
Methodology employed during UG/PG			
Cohort study	1 (2.6%)	2 (9.1%)	3 (5.0%)
Cross-sectional study	21 (55.3%)	12 (54.5%)	33 (55%)
Qualitative study	6 (15.8%)	5 (22.7%)	11 (18.3%)
Other	10 (26.3%)	3 (13.7%)	13 (21.7%)
Funding available			
Self-funding	32 (84.2%)	18 (81.8%)	50 (83.3%)
External funding	2 (5.3%)	0 (0.0%)	2 (3.4%)
Other	4 (10.5%)	4 (18.2%)	8 (13.3%)
Did you submit for publication?			
No	31 (81.6%)	13 (59.0%)	44 (73.3%)
Yes	7 (18.4%)	9 (41.0%)	16 (26.7%)
Would you like to do research in the future?			
Yes	37 (97.3%)	22 (100.0%)	59 (98.3%)
No	1 (2.7%)	0 (0.0%)	1 (1.7%)

### Details of research dissertations of participants

We asked participants to give details of their research projects undertaken at intern and resident level. Interns (87%) were much more likely to have undertaken retrospective research than residents (14%) (Table 2) which is in keeping with academic regulations in Rwanda which require residents to undertake prospective data collection. Interns were much more likely to undertake research in tertiary sites (87%). Cross-sectional studies were the most commonly undertaken methodology, being performed by 55% of participants. More than 80% of interns and residents reported that they “self-funded” their research. They therefore received no external financial backing to cover costs for travel, data collection, statistical analysis etc. The overall number (27%) of those who submitted their research for publication was low with only 18 and 41% of interns and residents respectively submitting for publication. Despite this, almost all respondents (98%) were interested in undertaking research in the future.

**Table 2:** Educational benefit of research – Likert responses^a^

	Group 1: Interns (*n* = 38) Mean	Group 2: Residents (*n* = 22) Mean	Total (*n* = 60) Mean	*p*-value and z-statistic*
To train medical students/residents how to conduct research/scholarship	4.82	4.73	4.78	*p* = 0.320 (z = − 0.99)
To expose medical students/residents to research	4.84	4.64	4.77	*p* = 0.027 (z = −2.206)
To improve patient care	4.86	4.86	4.75	*p* = 0.306 (z = −1.03)
To encourage medical students/residents to develop self-confidence	4.68	4.50	4.62	*p* = 0.411 (z = −0.82)
To develop skills in working independently	4.68	4.41	4.58	*p* = 0.093 (z = −1.68)
To encourage medical students/residents to pursue subspecialty fellowship	4.68	4.23	4.52	*p* = 0.008 (z = −2.63)
For scientific discovery	4.68	4.00	4.43	*p* = 0.003 (z = −2.97)
To teach medical students/residents problem-solving skills	4.39	4.36	4.38	*p* = 0.372 (z = −0.89)
To encourage medical students/residents to pursue careers in academic medicine	4.68	3.86	4.38	*p* < 0.001 (z = −4.12)
To give all medical students/residents the experience of submitting an institutional review board (IRB) ethics application	4.11	4.18	4.13	*p* = 0.961 (z = −0.05)
Total educational benefit survey score (10-items)^b^	46.3 (±4.6)	44.1 (±4.6)	45.5 (±4.7)	*p* = 0.023 (z = −2.27.)

^a^Likert questions used and scoring: Extremely important = 5; Very Important = 4; Moderately important = 3; Slightly important = 2; Not at all important =1; *Mann-Whitney test; ^b^Cronbach reliability = 0.865; ±Standard deviation

### Educational benefit of research

Both groups acknowledged the educational importance of undertaking research with “training how to conduct research” (mean = 4.78) being perceived as the most important educational benefit (Table 2). Experience of the IRB ethics application was perceived as the lowest educational benefit (mean 4.13). The combined survey scores revealed that interns reported a significantly higher educational benefit score than residents (46.3 versus 44.1, *p* = 0.023).

### Developing skills for publishing research work

Both groups acknowledged the importance of undertaking research to develop skills for publishing research work (Table 3). Interestingly, both groups also recognized that their research experience was valuable for better understanding of published works.

**Table 3:** Developing skills for publishing research work - Likert responses^a^

	Group 1: Interns (*n* = 38) Mean	Group 2: Residents (*n* = 22) Mean	Total (*n* = 60) Mean	*p*-value and z-statistic*
To better understand published works (evidence-based medicine)	4.68	4.55	4.63	*p* = 0.152 (z = − 1.43)
To have all medical students/residents submit their work for presentation at a regional/national conference	4.05	4.09	4.07	*p* = 0.864 (z = −0.17)
To have all medical students/residents present their scholarly project locally	4.13	3.82	4.02	*p* = 0.111 (z = − 1.60)
To have all medical students/residents submit their work to a peer-reviewed journal	3.97	4.09	4.02	*p* = 0.789 (z = −0.27)
Total publication survey score (4-items)^b^	16.8 (±3.2)	16.5 (±2.5)	16.7 (±3.0)	*p* = 0.394 (z = −0.85)

^a^Likert questions used and scoring: Extremely important = 5; Very Important = 4; Moderately important = 3; Slightly important = 2; Not at all important =1; *Mann-Whitney test; ^b^Cronbach alpha reliability = 0.820; ±Standard deviation

### Barriers to research

Both groups acknowledged the challenges of undertaking research (Table 4). Four of the five most significant perceived barriers to research were external to the students own control, namely: faculty lacking time, lack of funding, lack of statistical support, and lack of faculty experienced in conducting research. “Lack of time for faculty to mentor students/residents” (mean = 4.40) was perceived as the most significant challenge to undertaking research in Rwanda. Gaining approval at the IRB was perceived as the lowest barrier to conducting research in Rwanda. There was no significant difference in the perceived barriers between interns and residents on the barrier survey scale. Those students who had previously submitted research for publication (*n* = 16) perceived fewer barriers to research (45.0 versus 50.7, *p* = 0.064) (Table 5).

**Table 4:** Barriers to research – Likert responses^a^

	Group 1: Interns (*n* = 38) Mean	Group 2: Residents (*n* = 22) Mean	Total (*n* = 60) Mean	*p*-value and z-statistic*
Lack of time for faculty to mentor students/residents	4.45	4.32	4.40	*p* = 0.369 (z = −0.90)
Lack of funding to support medical students/residents conducting research	4.50	3.55	4.15	*P* < 0.001 (z = −4.37)
Lack of personal knowledge of research process	4.05	3.55	4.00	*p* = 0.027 (z = −2.21)
Lack of statistical support	4.24	3.55	3.98	*p* = 0.003 (z = − 3.02)
Lack of faculty experienced in conducting research	3.95	4.00	3.97	*p* = 0.910 (z = −0.11)
Lack of time to conduct scholarly activity	4.08	3.55	3.88	*p* = 0.009 (z = −2.62)
Lack of rewards or motivations	3.92	3.55	3.78	*p* = 0.072 (z = − 1.80)
Lack of proper laboratory and other facilities	3.89	3.55	3.77	*p* = 0.053 (z = − 1.94)
Medical student/resident attitudes toward conducting research	3.84	3.55	3.73	*p* = 0.194 (z = − 1.30)
Burden of other educational activities (e.g. exams and clinical rotations)	3.79	3.55	3.70	*p* = 0.170 (z = − 1.37)
More interested in clinical activities	3.24	3.55	3.35	*p* = 0.390 (z = −0.86)
See no personal future in research	3.18	3.55	3.32	*p* = 0.147 (z = −1.45)
Difficulty of obtaining approval by Institution review board (ethics)	3.08	3.55	3.25	*p* = 0.106 (z = −1.62)
Barrier to research survey scale (13-items)^b^	50.2 (±8.1)	47.3 (±9.2)	49.2 (±8.6)	*p* = 0.509 (z = −0.66)

^a^Likert questions used and scoring: Strongly agree = 5; Agree = 4; Neither agree nor disagree = 3; Disagree = 2; Strongly disagree =1; *Mann-Whitney test; ^b^Cronbach reliability = 0.888; ±Standard deviation

**Table 5:** Perceptions of those who had submitted research for publication

Did you submit your research for publication	Yes (*n* = 16)	No (*n* = 44)	*p*-value and z-statistic
Educational benefits score	45.1 (±4.2)	45.6 (±4.9)	*p* = 0.433 (z = − 0.78)
Publishing score	16.3 (±2.8)	16.9 (±3.1)	*p* = 0.375 (z = − 0.89)
Barriers to research score	45.0 (±10.1)	50.7 (±7.5)	*p* = 0.064 (z = − 1.85)

### Reliability of the responses to the questionnaire

The questionnaire was reviewed by local and senior academics to ensure content validity. The internal consistency (Cronbach alpha) of the responses to the “educational benefits” (10-item), “publishing” (4-item) and “barriers” (13-items) sections were 0.87, 0.82 and 0.89 respectively.

## Discussion

The objective of this study was to describe the perceived attitudes towards the educational benefits and barriers regarding research activity and to describe the research methods used by interns and residents in Rwanda.

### Educational benefits of research for interns and residents

98% of participants reported being interested in undertaking future research. It is therefore not surprising that the highest perceived educational benefit was to train students how to conduct research. Both interns and residents showed positive attitudes toward research. Similar findings have been documented in the literature [7]. However, there is a documented lower level of positive attitudes toward research in developing countries [26].

### Developing skills for publishing research work

In order to disseminate research, students need to develop the necessary skills involved, namely how to write scientifically and concisely. The participants here all agreed that it was very important to have all medical students/residents submit their work at conferences or in peer-reviewed journals. Respondents felt that conferences or local dissemination were more important than peer-reviewed journals (Table 3). There is evidence in Rwanda that active engagement with faculty and students, via workshops, will lead to an increase in the dissemination of research results [27].

### Barriers to research

One finding in our study is that students perceived a “Lack of faculty experienced in conducting research”. In Rwanda, all residents undertake research, therefore all faculty who have undertaken speciality training in Rwanda, should have experience in undertaking research. Therefore, this perceived lack of faculty experience is most likely to reflect students’ perceptions that faculty lack the time to supervise them rather than the necessary skills. Faculty lacking time to supervise research may be explained by their busy teaching schedule, demands from other academic activities, and few faculty in each department. In Ontario, Canada it was found that the most significant barriers to involvement in research in medical school were time, availability of research mentors, formal teaching of research methodology, and the perception that the student would not receive appropriate acknowledgment for work put towards a research project [9]. In Uganda, both interns and residents reported a lack of collaborations, funds, facilities, knowledge, and guidance as barriers to research for them [5]. In a study done in Iran, lack of funding support was noted as the primary barrier to research amongst medical students [23].

### Research undertaken in Rwanda by interns and residents

Cross-sectional research is the most common methodology performed by Rwandan interns and residents. This is understandable as most research undertaken by interns and residents is time-bound during the course of studies. Cross-sectional research is an appropriate “toe-in-the-water” for students and gives an opportunity to gain experience in the research process despite multiple-barriers. Interns undertook their research at teaching-hospital sites in a retrospective manner. This is again understandable in the context of the barriers that they face in undertaking research in this setting in terms of financial cost and logistics.

### Students submitting for publication

We found that only 27% of the students reported that they had submitted their research work for publication. Researchers in Rwanda should remain committed to submitting the results of all human research they conduct for publication in a peer-review journal. *Research* is dependent on the willingness of participants to expose themselves to the risks involved [28]. The ethical justification for these risks is that society will eventually benefit from the knowledge gained from the study. Researchers, therefore, have an ethical responsibility to report the results of research involving human subjects [29, 30]. The Declaration of Helsinki (2014) states that “Researchers, authors, sponsors, editors and publishers all have ethical obligations with regard to the publication and dissemination of the results of research” [31]. The future synthesis of research in systematic reviews is also compromised by dissemination bias if journal publications represent a biased selection of all studies that have been conducted [32]. In the case of Rwandan interns and residents, many may still lack the skills required to overcome the barriers in navigating the peer-review process and therefore supervisors should provide significant support in achieving this goal.

### Limitations

The overall response rate was 30%, with 20 and 54% of interns and residents responding respectively. A study of 1607 questionnaires in the social sciences found an average response rate of 57% [33]. The lower response rate in our interns may reflect that the contact details were not up to date as they had moved on from their studies. The participants who took part may have more interest in research activities and were therefore more inclined to take part. This form of response bias could account for some of our findings. Due to the anonymous nature of the questionnaire we have no comparison data on the non-responders. The questionnaire was electronically administered which could have biased the results to electronically-fluent participants and those with internet credit. The responses were limited to Likert format, and therefore qualitative research on the topic could reveal a deeper understanding of the perceptions and attitudes of our subjects. There may have been a tendency for questionnaire respondents to give positive answers to the questions (acquiescence bias), however, the responses were anonymized to reduce the risk of participants giving answers they believed the researchers wanted. Subjects were given a small payment for participation reflective of the time to complete the questionnaire and this could have introduced some bias.

### Application of the results

It is an aim of the University of Rwanda (UR) to improve the quality and quantity of their research output. The results of this study are therefore relevant to other institutions in the East African community with similar aspirations. The responses in this survey demonstrate that medical students and residents look positively on research activities, but they still face barriers. Time needs to be allocated to students in the academic calendar to work on their research activities. Assigning time to faculty to teach and mentor students in research is important if they are going to undertake significant research. This is challenging in resource-limited settings such as the UR where the faculty to student ratio is low. This has recently been addressed in Rwanda with non-academic clinicians at teaching hospitals being contractually required to engage in teaching and learning activities.

### Reliability of the questionnaire

The internal consistency of the responses to the “educational benefits” (α = 0.87), “publishing” (α = 0.82) and “barriers” (α = 0.89) sections were good. This shows that the respondents in this study responded consistently within each concept. The Cronbach was also not too high (> 0.95), which would suggest redundant items [34]. No formal assessment of validity was undertaken, except for review of content validity by four faculty members. Despite this, the questionnaire could make a useful tool for other academic departments wanting to investigate the attitudes and perceptions of their cohort of students. Although not tested in this study, the questionnaire has the potential to be used as a mentoring tool, with supervisors assessing the research needs of their individual students prior to embarking on the research journey.

## Conclusions

This is the first study to assess attitudes and perceptions to research amongst Rwandan medical students and residents. This has been done with a valid questionnaire, which has demonstrated consistent data. Both interns and residents understood the importance of research but they both still face barriers which are mostly out of their control. These barriers impact how and whether they conduct research. The institutions in charge should take measures in order to establish effective solutions to these barriers. The results should be used to strengthen research activities in Rwanda and further afield in the East African region.

## Abbreviations

CMHS: College of Medicine and Health Sciences
HLI: Higher Learning Institutions
HRH: Human Resources for Health
IRB: Institutional Review Board
MMED: Masters in medicine
PGs: Postgraduates
STROBE: Strengthening the Reporting of Observational Studies in Epidemiology
UGs: Undergraduates
UR: University of Rwanda
UR: University of Rwanda
WHO: World Health Organization
